# Common variants in *SIRT1* and human longevity in a Chinese population

**DOI:** 10.1186/s12881-016-0293-3

**Published:** 2016-04-18

**Authors:** Rong Lin, Dongjing Yan, Yunxia Zhang, Xiaoping Liao, Gu Gong, Junjie Hu, Yunxin Fu, Wangwei Cai

**Affiliations:** Department of Biology, Hainan Medical College, Haikou, 571199 Hainan China; Department of Biochemistry and Molecular Biology, Hainan Medical College, Haikou, 571199 Hainan China; Department of Neurology, the Affiliated Hospital of Hainan Medical College, Haikou, 571199 Hainan China; College of Agriculture, Hainan University, Haikou, 570228 Hainan China; Division of Biostatistics and Human Genetics Center, The University of Texas Health Science Center at Houston, 1200 Herman Pressler, Houston, TX 77025 USA; Laboratory for Conservation and Utilization of Bio-Resources, Yunnan University, Kunming, 650091 Yunnan China

**Keywords:** *SIRT1*, Case–control association design, Human longevity, Single nucleotide polymorphism

## Abstract

**Background:**

The silent information regulator *SIR2*/*SIRT1*gene has been demonstrated as regulating lifespan in many model organisms, including yeast, worms, fruit flies and rodents. *SIRT1*, the human homolog of *SIR2*, is considered a candidate gene as a modifier of human life expectancy.

**Methods:**

In the current study we included 616 long-lived individuals and 846 matched younger controls to investigate associations between 8 common single nucleotide polymorphisms (SNPs) (i.e., rs12778366, rs3758391, rs3740051, rs33957861, rs7896005, rs12413112, rs11599176 and rs4746720) in the *SIRT1* gene and human longevity.

**Results:**

The 8 SNPs had strong linkage disequilibrium (LD) and were in an LD block, which was characterized by 4 common haplotypes that capture 99.3 % of the genetic variation present within it. We found no evidence for statistically significant associations between the tested *SIRT1* SNPs and longevity at the allele, genotype or haplotype levels.

**Conclusions:**

Current findings show that several common variants in *SIRT1* are not associated with human longevity.

**Electronic supplementary material:**

The online version of this article (doi:10.1186/s12881-016-0293-3) contains supplementary material, which is available to authorized users.

## Background

Increased expression of silent information regulator 2 (*SIR2*) are associated with extended lifespan of lower organisms such as yeast [1], fruit flies [2] and worms [3]. Out of seven identified mammalian homologues, Sirtuin 1 (SIRT1) is the most similar to SIR2 [4, 5].

SIRT1 is a nicotinamide adenine dinucleotide-dependent histone deacetylase [6], and could influence life span in several ways. It has been shown that SIRT1 has an effect on fat metabolism [7]. SIRT1 protein upregulated through food withdrawal was found to bind to and repress the fat regulator peroxisome proliferator-activated receptor-γ (PPAR-γ) in murine adipocytes, thus stimulating fat breakdown. Because a reduction in fat storage in white adipose tissue is a primary way by which calorie restriction (CR) extends lifespan in mammals, these results suggest that activation of SIRT1 mediated by energy restriction could be a possible molecular mechanism of mammalian lifespan regulation.

Furthermore, the effect of SIRT1 on mammalian longevity may be exerted partly through its association with insulin signaling, which has been shown to increase lifespan in fat-specific insulin receptor knockout mice [8]. Activation of SIRT1 also improves glucose tolerance and enhances insulin secretion in response to glucose in pancreatic β cells [9, 10]. SIRT1 upregulation also promotes hepatic gluconeogenesis and inhibits glycolysis through peroxisome proliferator-activated receptor γ coactivator 1-α (PGC1-α) during fasting [11]. These findings suggest that increased SIRT1 activity results in a favorable metabolic profile for long life.

In addition, the role of SIRT1 in providing resistance to damage- or stress-induced apoptosis may help to preserve organ function over time and favor long lifespan under certain environmental conditions, such as CR [12–15]. Recent evidence also suggests one way in which SIRT1 may increase organismal longevity is by its protective activity against neuronal degeneration [16–19].

The involvement of *SIRT1* in human life span has been previously studied in a case–control study of long-lived and younger individuals [20–22]. The findings, however, are controversial and do not confirm whether *SIRT1* gene has an influence on human life span. Flachsbart et al. did not observe any differences in *SIRT1* allele and haplotype frequencies between the long-lived and younger Caucasians [20]. Kim et al. found that the minor allele frequency of the *SIRT1* single nucleotide polymorphism (SNP) rs7896005 was higher in the long-lived Caucasians than in the young Caucasians [21]. SNP rs4746720 has been shown to be significant associated with human longevity in a Chinese population from Yongfu region of Guangxi [22].

The inconsistent observations warrant more examination of the role of *SIRT1* gene in human longevity. Therefore, in this study, we attempted to analyze the association between common variations in the *SIRT1* gene and human longevity in a Chinese population from Hainan Island.

## Methods

### Study population

A total of 1,462 unrelated Chinese subjects were included in the present study: 616 long-lived individuals (LLIs) and 846 controls. All LLIs were ≥ 98 years of age at the time of recruitment (mean age: 102.4 ± 2.3 years, 38 Li and 578 Han people, 102 males and 514 females). The gender ratio in the LLIs was 83.4 % females vs. 16.6 % males, and 93.8 % of LLIs was Han Chinese. The control subjects were 30–70 years old (mean age: 48.9 ± 10.6 years, 69 Li and 777 Han people, 159 males and 687 females) and matched the LLIs by gender, ethnical ancestry and geographical origin in the Hainan Island. Based on the sixth national population census database of the People’s Republic of China in 2010, among China’s 31 provinces, autonomous regions and municipalities, the largest number of centenarians per 10,000 inhabitants aged 65 years or over is in Hainan (16.64), followed by Guangxi (7.00), Guangdong (6.07), Xinjiang (5.25), Shanghai (3.98) [23]. All subjects gave informed consent and the study was approved by the Ethics Committee of Hainan Medical College and by the local data protection authorities.

### SNP selection and genotyping

Six SNPs (rs12778366, rs3758391, rs3740051, rs7896005, rs10823107 and rs4746720) were selected from the phase II HapMap Han Chinese (CHB) population [HapMap release 27 (Feb 2009), NCBI Build 36], and are able to tag 82 common SNPs of the *SIRT1* gene and its 5 kb up-/downstream region (chromosome 10: 69309433..69353147 43.72kbp) with *r*^2^ greater than 0.90 and minor allele frequency (MAF) ≥0.05 (Additional file 1 Table S1). Other three common SNPs (i.e., rs33957861, rs12413112 and rs11599176) were selected from the 1000 Genomes Project database.

The genotypes of all SNPs were determined, blind to subject status, using a custom-by-design 48-Plex SNPscan™ Kit (Cat#:G0104; Genesky Biotechnologies Inc., Shanghai, China), which was developed according to patented SNP genotyping technology by Genesky Biotechnologies Inc.. As described by Chen et al. [24], it was based on double ligation and multiplex fluorescence polymerase chain reactions.

Finally, 8 out of 9 SNPs were successfully genotyped; however, tag SNP rs10823107 failed in genotyping and was removed from the analysis. This SNP did not tag any other SNPs at *r*^2^ greater than 0.9 in the CHB population (Additional file 1 Table S1). The genotyping success rates were more than 99 % (Table 1) and the concordance rates were more than 99 % based on 5.3 % duplicate samples. No significant deviation from Hardy-Weinberg equilibrium (HWE) was observed for all SNPs in the controls (*P* > 0.05) (Table 1).

**Table 1:** Primary information for single-nucleotide polymorphisms (SNPs) genotyped

	SNPs	Chromosome position	SNP location in gene	Major > Minor allele	Call rate	HWE (control)
1	rs12778366^a^	chr10:69313085	5′ upstream	T > C	100 %	0.55
2	rs3758391^a^	chr10:69313348	5′ upstream	T > C	99.86 %	1
3	rs3740051^a^	chr10:69313965	5′ upstream	A > G	99.79 %	0.8
4	rs33957861	chr10:69316982	Intron	C > T	99.93 %	0.9
5	rs7896005^a^	chr10:69321131	Intron	A > G	99.93 %	0.89
6	rs12413112	chr10:69321872	Intron	G > A	100 %	1
7	rs11599176	chr10:69323781	Intron	A > G	99.93 %	1
	rs10823107^a,b^	chr10:69330230	Intron			
8	rs4746720^a^	chr10:69346836	3′ untranslate region	T > C	100 %	0.074

HWE indicates Hardy-Weinberg equilibrium

^a^Tag SNPs selected from the HapMap database

^b^SNPs which failed in genotyping and was removed from the analysis

### Statistical analysis

SNPStats (http://bioinfo.iconcologia.net/SNPstats_web), which is a web-based software tool, was used for all analyses. All *P* values presented in this study are two-sided, and *P* ≤ 0.05 was used as threshold of statistical significance. Departure from HWE of each SNP frequency was assessed using an exact test in control subjects. Odds ratios (ORs) and 95 % confidence intervals (CIs) were calculated to describe the strength of association between certain SNPs and human longevity.

The D’ and *r*^2^ statistics were determined to represent linkage disequilibrium (LD), and a D’ value of ≥0.8 indicated the related SNPs formed one block. The association parameters of human longevity were estimated for each haplotype by comparison with the most frequent haplotype. Effects associated with rare haplotypes (frequency <0.5 %) were estimated after combining them as one. Finally, the SHEsis software (http://analysis.bio-x.cn/myAnalysis.php) was also adopted to yield similar haplotype block structures, D’ and *r*^2^ compared with SNPStats.

## Results

**Fig. 1:**
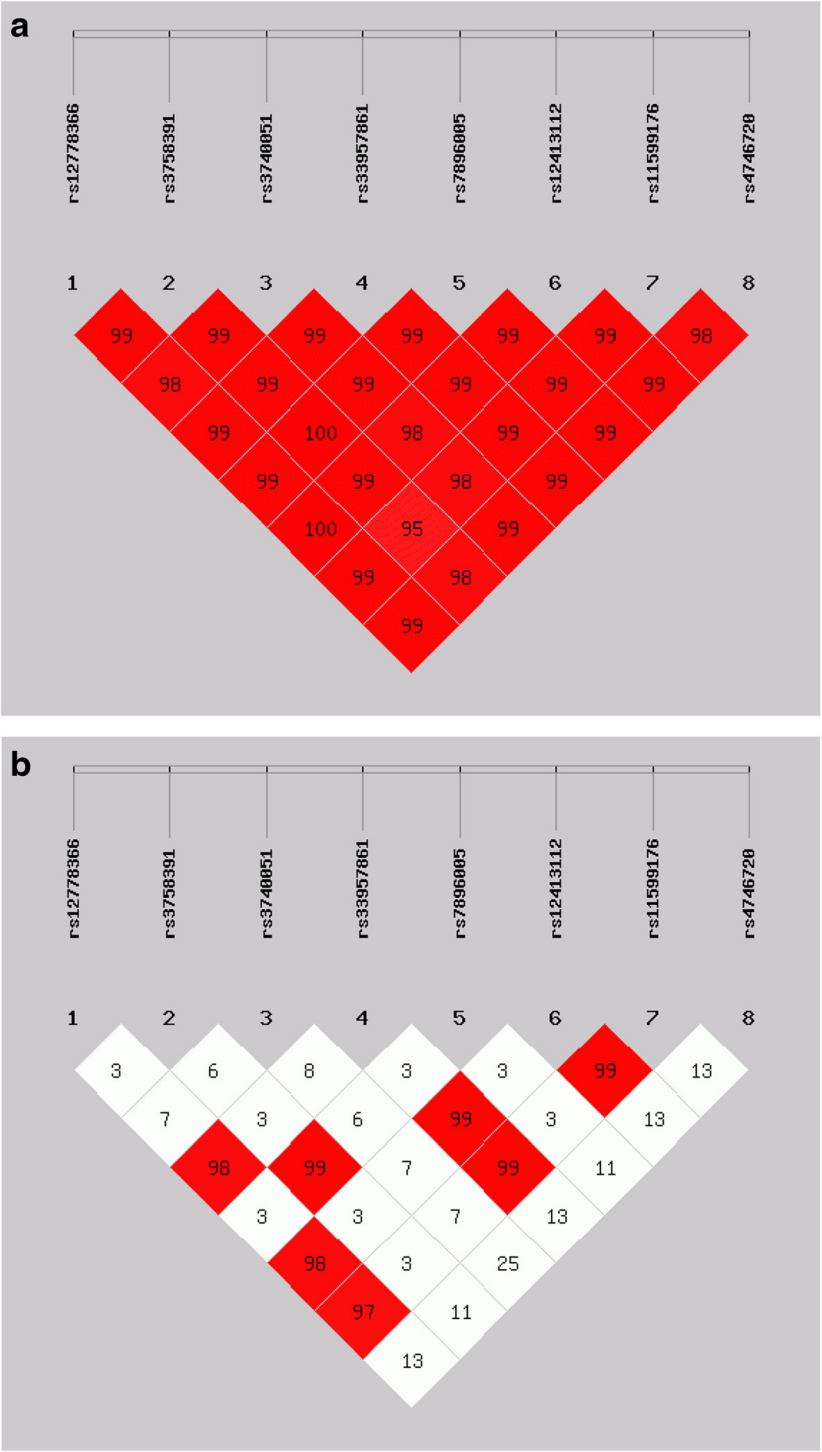
Pairwise linkage disequilibrium (LD) of the 8 SNPs in the *SIRT1* gene under study. LD was measured by the D’ and *r* ^2^statistics using the data from all subjects. The colors indicate the strength of pairwise LD. The darker color the higher the D’ or *r* ^2^. **a**. D’ values **b**. *r* ^2^ values Notes: For D’, a value of 100 reflects complete dependency between markers; For *r* ^2^, a value of 100 reflects perfect dependency between markers. (SHEsis Software, ver. online)

No significant differences in genotype and allele distribution of the SNPs were observed between LLIs and younger controls (Table 2). All pairwise D’ values between the 8 SNPs were equal to or greater than 0.95 (Fig. 1a), which suggested that the 8 SNPs had strong LD and were in one block. Pairwise *r*^2^ values between SNP 1, 4, 6 and 7 (i.e., rs12778366, rs33957861, rs12413112 and rs11599176) were all greater than 0.95 (Fig. 1b), which indicated these 4 SNPs were almost in perfect LD. SNP 2 and 5 (i.e., rs3758391 and rs7896005) were also almost in perfect LD (D’ = 1, *r*^2^ = 0.99). Other pairwise *r*^2^ values were very low (all <0.25). As shown in Table 3, the eight SNPs constituted only four common haplotypes, which covered 99.3 % of the present Chinese population. None of the haplotypes differed significantly in frequency between the cases and the controls.

**Table 2:** Genotype and allele frequencies of *SIRT1* polymorphisms in the long-lived individuals and controls

Polymorphisms	Genotype/Allele	Case	Control	OR (95%CI)	*P*
rs12778366	T/T	427 (69.3 %)	578 (68.3 %)	1	0.64
	C/T	169 (27.4 %)	246 (29.1 %)	0.93 (0.74–1.17)	
	C/C	20 (3.2 %)	22 (2.6 %)	1.23 (0.66–2.28)	
	T	1023 (83.0 %)	1402 (82.9 %)	1	0.90
	C	209 (17.0 %)	290 (17.1 %)	0.99 (0.81–1.20)	
rs3758391	T/T	441 (71.6 %)	620 (73.5 %)	1	0.72
	C/T	161 (26.1 %)	207 (24.5 %)	1.09 (0.86–1.39)	
	C/C	14 (2.3 %)	17 (2 %)	1.16 (0.56–2.37)	
	T	1043 (84.7 %)	1447 (85.7 %)	1	0.42
	C	189 (15.3 %)	241 (14.3 %)	1.09 (0.88–1.34)	
rs3740051	A/A	310 (50.5 %)	436 (51.6 %)	1	0.90
	G/A	258 (42 %)	345 (40.8 %)	1.05 (0.85–1.31)	
	G/G	46 (7.5 %)	64 (7.6 %)	1.01 (0.67–1.52)	
	A	878 (71.5 %)	1217 (72.0 %)	1	0.76
	G	350 (28.5 %)	473 (28.0 %)	1.03 (0.87–1.21)	
rs33957861	C/C	425 (69.1 %)	578 (68.3 %)	1	0.91
	C/T	170 (27.6 %)	242 (28.6 %)	0.96 (0.76–1.21)	
	T/T	20 (3.2 %)	26 (3.1 %)	1.05 (0.58–1.90)	
	C	1020 (82.9 %)	1398 (82.6 %)	1	0.83
	T	210 (17.1 %)	294 (17.4 %)	0.98 (0.81–1.19)	
rs7896005	A/A	440 (71.5 %)	623 (73.6 %)	1	0.64
	G/A	161 (26.2 %)	207 (24.5 %)	1.10 (0.87–1.40)	
	G/G	14 (2.3 %)	16 (1.9 %)	1.24 (0.60–2.56	
	A	1041 (84.6 %)	1453 (85.9 %)		0.35
	G	189 (15.4 %)	239 (14.1 %)	1.10 (0.90–1.36)	
rs12413112	G/G	426 (69.2 %)	576 (68.1 %)	1	0.87
	G/A	170 (27.6 %)	244 (28.8 %)	0.94 (0.75–1.19)	
	A/A	20 (3.2 %)	26 (3.1 %)	1.04 (0.57–1.89)	
	G	1022 (83.0 %)	1396 (82.5 %)	1	0.75
	A	210 (17.0 %)	296 (17.5 %)	0.97 (0.80–1.18)	
rs11599176	A/A	423 (68.8 %)	576 (68.1 %)	1	0.92
	G/A	172 (28 %)	244 (28.8 %)	0.96 (0.76–1.21)	
	G/G	20 (3.2 %)	26 (3.1 %)	1.05 (0.58–1.90)	
	A	1018 (82.8 %)	1396 (82.5 %)	1	0.86
	G	212 (17.2 %)	296 (17.5 %)	0.98 (0.81–1.19)	
rs4746720	T/T	241 (39.1 %)	314 (37.1 %)	1	0.73
	C/T	271 (44 %)	382 (45.1 %)	0.92 (0.74–1.16)	
	C/C	104 (16.9 %)	150 (17.7 %)	0.90 (0.67–1.22)	
	T	753 (61.1 %)	1010 (59.7 %)	1	0.45
	C	479 (38.9 %)	682 (40.3 %)	0.95 (0.82–1.09)

**Table 3:** Association of *SIRT1* haplotypes with human longevity

Haplotype	rs12778366-rs3758391-rs3740051-rs33957861-rs7896005-rs12413112-rs11599176-rs4746720	Frequency		OR (95 % CI)	*P*
		Case	Control		
1	T-T-A-C-A-G-A-C	0.3864	0.4025	1	
2	T-T-G-C-A-G-A-T	0.2841	0.2784	1.06 (0.88–1.27)	0.54
3	C-T-A-T-A-A-G-T	0.1696	0.1708	1.03 (0.83–1.27)	0.78
4	T-C-A-C-G-G-A-T	0.1534	0.1413	1.13 (0.90–1.41)	0.29
rare		0.0065	0.0072	0.95 (0.31–2.90)	0.93
Global					0.87

The study recruited a small number of Li subjects (*n* = 107). In order to avoid false association due to population stratification, we also restricted analyses to those Han subjects (Additional file 1 Tables S2-S3) and the results were similar to those in all subjects. Furthermore, we performed gender-stratified analyses but did not identify positive association in women or men (Additional file 1 Tables S4-S5).

## Discussion

In this study, we tested the association between eight common variants in the *SIRT1* gene and human longevity in a Chinese population. No evidence for an association was detected between any of the tested SNPs and human longevity at the allele, genotype or haplotype levels. However, this does not exclude the possibility that low-frequency and rare variants in *SIRT1* as well as allelic variants in direct regulators or downstream substrates of *SIRT1* could play important roles in extending human lifespan.

A study by Flachsbart et al., in which five SNPs (rs3758391,rs1885472,rs2273773, rs10997870 and rs2234975) was analyzed, also did not detect any significant association between the tested SNPs and human longevity at the allele, genotype or haplotype levels in white individuals [20]. Similar to our result, the five SNPs comprised a single LD block with high pairwise D’ values (range from 0.92 to 1.00) and defined only five common haplotypes. Among them, rs3758391, rs1885472 and rs10997870 could be tagged by SNP 2 and 5 (rs3758391 and rs7896005) (all *r*^2^ = 1 in CHB, all *r*^2^ > 0.85 in Utah-Europeans (CEU)), rs2273773 by SNP 3 (rs3740051) (*r*^2^ = 0.929 and 1, respectively) in CHB and CEU populations, and rs2234975 is virtually monomorphic in CHB population.

In addition, in the study by Huang et al. [22], rs3758391 and rs10997870, which could be tagged by SNP 2 and 5 (rs3758391 and rs7896005) (all *r*^2^ = 1) in CHB population, also showed non-significant association with human longevity in Chinese. Similarly, in the study by Willcox et al. [25], rs7069102 and rs1885472, which could be tagged by SNP 2 and 5 (rs3758391 and rs7896005) in CHB and Japanese in Tokyo (JPT) populations (all *r*^2^ = 1), also showed non-significant association with human longevity in Japanese. However, in the study by Kim et al., the frequency of the A allele of SNP 5 (rs7896005) was higher in the long-lived Caucasians than in the young Caucasians [21] (Additional file 1 Table S6).

In this study, SNP 8 (rs4746720) was not associated with human longevity which in another study in Chinese by Huang et al. [22] demonstrated that the C/T genotype frequency in the cases is higher than in the controls. In a word, the result of SNP 5 and 8 are inconsistent (Additional file 1 Table S6). To date, there are only 5 non-genome-wide association studies that investigated variants in *SIRT1* in relation to human longevity (the present study and 4 published), which we all listed in Additional file 1 Table S6. Only two studies have found significant association of variants in *SIRT1* with human longevity.

Furthermore, despite the strong evidence in favor of a role for *SIRT1* on lifespan, none of the genetic variants of this gene have been genome-wide significantly associated with longevity [26–33]. Therefore it is possible that genes that regulate SIRT1, rather than *SIRT1* itself, are under demographic pressure and, hence, better targets to extend lifespan. For example, calcium/calmodulindependent protein kinase IV (CAMKIV), which activates SIRT1 protein, and one *CAMKIV* loci (i.e., rs10491334) have been identified to be associated with longevity at a genome-wide level [29]. Thus, genes that interact with SIRT1 and potential gene-gene interaction between them must be taken into account.

To be noted, all 4 published non-genome-wide association studies [20–22, 25] and the present study as well as the genome-wide association studies on longevity [26–33] focused on common variants and did not include low-frequency and rare variants. As longevity in the general population is quite rare, longevity may be regulated by low-frequency and rare variants, rather than common variants. Future genetic studies should consider performing association analyses for low-frequency and rare variants, especially for coding non-synonymous variants.

In the present study, the 8 SNPs successfully genotyped were able to capture 81 of 106 (76.4 %) and 24 of 25 (96.0 %) common SNPs across the *SIRT1* gene and its 5 kb up-/downstream region in the 1000 Genomes and HapMap Project databases, respectively, at *r*^2^ greater than 0.9. More variants are cataloged in 1000 Genomes than in HapMap. But not all variants cataloged in HapMap are also cataloged in 1000 Genomes and 99 % of HapMap common variants were found in 1000 Genomes [34]. Both the HapMap and 1000 Genomes Project databases are useful resources for human genetics. It is best to use the two databases to pick tag SNPs for a genetic association study. However, the present study selected tag SNPs from the HapMap Project database and only included three common SNPs from the 1000 Genomes Project database. We recognized that it was a limitation of the study. More variants in 1000 Genomes are required to be estimated.

## Conclusions

In summary, this study showed that several common variants, which span across the *SIRT1* gene and its 5 kb up-/downstream region, are not related to longevity in Chinese. More studies are needed to confirm the findings and further clarify the role of *SIRT1* and its regulators in human longevity.

## Additional file

Additional file 1: Table S1. Details for single-nucleotide polymorphisms (SNPs) tagged by genotyped SNPs. **Table S2.** Genotype and allele frequencies of *SIRT1* polymorphisms in the Chinese Han long-lived individuals and controls. **Table S3.** Association of *SIRT1* haplotypes with human longevity in the Chinese Han long-lived individuals and controls. **Table S4.** Genotype and allele frequencies of *SIRT1* polymorphisms in the long-lived individuals and controls when stratified by gender. **Table S5.** Association of *SIRT1* haplotypes with human longevity when stratified by gender. **Table S6.** Association studies of *SIRT1* with human longevity. (DOC 226 kb)
