# Supplementary material for: Common variants in SIRT1 and human longevity in a Chinese population
Source: BMC Med Genet. 2016 Apr 18;17:31. doi: 10.1186/s12881-016-0293-3 (PMC4836161; doi:10.1186/s12881-016-0293-3)
Supplement: Additional file 1: Table S1. — Details for single-nucleotide polymorphisms (SNPs) tagged by genotyped SNPs. Table S2. Genotype and allele frequencies of SIRT1 polymorphisms in the Chinese Han long-lived individuals and controls. Table S3. Association of SIRT1 haplotypes with human longevity in the Chinese Han long-lived individuals and controls. Table S4. Genotype and allele frequencies of SIRT1 polymorphisms in the long-lived individuals and controls when stratified by gender. Table S5. Association of SIRT1 haplotypes with human longevity when stratified by gender. Table S6. Association studies of SIRT1 with human longevity. (DOC 226 kb) [file 12881_2016_293_MOESM1_ESM.doc]

**Additional file 1**

| Table S1. Details for single-nucleotide polymorphisms (SNPs) tagged by genotyped SNPs | | |
| --- | --- | --- |
|  | SNPs | Alleles captured |
| 1 | rs12778366^a^ | rs12778366,rs145758730,rs113045253,rs33957861,rs12413112,rs7075943,rs35598722,rs11599176,rs12783242,rs11595798,rs35689145,rs35695055,rs34104788,rs3818291,rs34971557 |
| 2 | rs3758391^a^ | rs61857476,rs4746716,rs10997853,rs10997854,rs10997855,rs10823102,rs12250285, rs3758391,rs932658,rs2394443,rs10997860,rs34416841,rs41299232,rs2236318,rs33960442,rs10823103,rs7896005,rs7904945,rs7904714,rs12252486,rs1885472,rs10997865,rs10823106,rs11596401,rs7095373,rs10997866,rs33955981,rs35620729,rs7069102,rs10997868,rs2224573,rs10997870,rs12242965,rs11594238,rs7073231,rs10823110,rs17526356,rs10823111,rs7091896,rs10823115,rs1467568,rs10997875 |
| 3 | rs3740051^a^ | rs10823100,rs3740051,rs3740053,rs737477,rs2236319,rs10997863,rs10823104,rs34097879,rs10823108,rs7096385,rs2273773,rs3818292,rs10823109,rs10823112 |
| 4 | rs33957861 | rs12778366,rs145758730,rs113045253,rs33957861,rs12413112,rs7075943,rs35598722,rs11599176,rs12783242,rs11595798,rs35689145,rs35695055,rs34104788,rs3818291, rs34971557 |
| 5 | rs7896005^a^ | rs61857476,rs4746716,rs10997853,rs10997854,rs10997855,rs10823102,rs12250285,rs3758391,rs932658,rs2394443,rs10997860,rs34416841,rs41299232,rs2236318,rs33960442,rs10823103,rs7896005,rs7904945,rs7904714,rs12252486,rs1885472,rs10997865,rs10823106,rs11596401,rs7095373,rs10997866,rs33955981,rs35620729,rs7069102,rs10997868,rs2224573,rs10997870,rs12242965,rs11594238,rs7073231,rs10823110,rs17526356,rs10823111,rs7091896,rs10823115,rs1467568,rs10997875 |
| 6 | rs12413112 | rs12778366,rs145758730,rs113045253,rs33957861,rs12413112,rs7075943,rs35598722,rs11599176,rs12783242,rs11595798,rs35689145,rs35695055,rs34104788,rs3818291, rs34971557 |
| 7 | rs11599176 | rs12778366,rs145758730,rs113045253,rs33957861,rs12413112,rs7075943,rs35598722,rs11599176,rs12783242,rs11595798,rs35689145,rs35695055,rs34104788,rs3818291, rs34971557 |
|  | rs10823107^a,b^ | rs10823107 |
| 8 | rs4746720^a^ | rs12571880,rs79269275,rs35480510,rs34294800,rs12570807,rs33992752,rs35416150,rs16924945,rs74790878,rs4746720 |
| ^a^Tag SNPs selected from the HapMap database. | | |
| ^b^SNPs which failed in genotyping and was removed from the analysis. | | |
| The 8 SNPs successfully genotyped were able to capture 81 common SNPs of the *SIRT1* gene and its 5 kb up-/downstream region (chromosome 10: 69309433..69353147 43.72kbp, NCBI Build 36) at *r*^2^ greater than 0.90 in CHB population (data from the 1000 Genomes Project). | | |

| Table S2. Genotype and allele frequencies of *SIRT1* polymorphisms in the **Chinese Han** long-lived individuals and controls. | | | | | |
| --- | --- | --- | --- | --- | --- |
| Polymorphisms | Genotype/Allele | Case | Control | OR(95%CI) | *P* |
| rs12778366 | T/T | 406 (70.2%) | 536 (69%) | 1.00 | 0.52 |
|  | C/T | 155 (26.8%) | 224 (28.8%) | 0.91 (0.72-1.16) |
|  | C/C | 17 (2.9%) | 17 (2.2%) | 1.32 (0.67-2.62) |
|  | T | 967(83.7%) | 1296(83.4%) | 1.00 |  |
|  | C | 189(16.3%) | 258(16.6%) | 0.98 (0.80-1.21) | 0.86 |
| rs3758391 | T/T | 407 (70.4%) | 569 (73.4%) | 1.00 | 0.47 |
|  | C/T | 157 (27.2%) | 190 (24.5%) | 1.16 (0.90-1.48) |
|  | C/C | 14 (2.4%) | 16 (2.1%) | 1.22 (0.59-2.53) |
|  | T | 971(84.0%) | 1328(85.7%) | 1.00 |  |
|  | C | 185(16.0%) | 222(14.3%) | 1.14 (0.92-1.41) | 0.23 |
| rs3740051 | A/A | 292 (50.7%) | 402 (51.8%) | 1.00 | 0.92 |
|  | G/A | 239 (41.5%) | 314 (40.5%) | 1.05 (0.84-1.31) |
|  | G/G | 45 (7.8%) | 60 (7.7%) | 1.03 (0.68-1.56) |
|  | A | 823(71.4%) | 1118(0.72) | 1.00 |  |
|  | G | 329(28.6%) | 434(28.0%) | 1.03 (0.87-1.22) | 0.73 |
| rs33957861 | C/C | 404 (70%) | 535 (68.8%) | 1.00 | 0.7 |
|  | C/T | 156 (27%) | 223 (28.7%) | 0.93 (0.73-1.18) |
|  | T/T | 17 (3%) | 19 (2.5%) | 1.18 (0.61-2.31) |
|  | C | 964(83.5%) | 1293(83.2%) | 1.00 |  |
|  | T | 190(16.5%) | 261(16.8%) | 0.98 (0.79-1.20) | 0.82 |
| rs7896005 | A/A | 406 (70.4%) | 572 (73.6%) | 1.00 | 0.4 |
|  | G/A | 157 (27.2%) | 190 (24.4%) | 1.16 (0.91-1.49) |
|  | G/G | 14 (2.4%) | 15 (1.9%) | 1.31 (0.63-2.75) |
|  | A | 969(84.0%) | 1334(85.8%) | 1.00 |  |
|  | G | 185(16.0%) | 220(14.2%) | 1.16 (0.94-1.43) | 0.18 |
| rs12413112 | G/G | 405 (70.1%) | 534 (68.7%) | 1.00 | 0.67 |
|  | G/A | 156 (27%) | 224 (28.8%) | 0.92 (0.72-1.17) |
|  | A/A | 17 (2.9%) | 19 (2.5%) | 1.18 (0.61-2.30) |
|  | G | 966(83.6%) | 1292(83.1%) | 1.00 |  |
|  | A | 190(16.4%) | 262(16.9%) | 0.97 (0.79-1.19) | 0.77 |
| rs11599176 | A/A | 402 (69.7%) | 534 (68.7%) | 1.00 | 0.74 |
|  | G/A | 158 (27.4%) | 224 (28.8%) | 0.94 (0.74-1.19) |
|  | G/G | 17 (3%) | 19 (2.5%) | 1.19 (0.61-2.32) |
|  | A | 962(83.4%) | 1292(83.1%) | 1.00 |  |
|  | G | 192(16.6%) | 262(16.9%) | 0.98 (0.80-1.21) | 0.88 |
| rs4746720 | T/T | 226 (39.1%) | 282 (36.3%) | 1.00 | 0.53 |
|  | C/T | 256 (44.3%) | 354 (45.6%) | 0.90 (0.71-1.14) |
|  | C/C | 96 (16.6%) | 141 (18.1%) | 0.85 (0.62-1.16) |
|  | T | 708(61.2%) | 918(59.1%) | 1.00 |  |
|  | C | 448(38.8%) | 636(40.9%) | 0.92 (0.79-1.07) | 0.27 |

| Table S3. Association of *SIRT1* haplotypes with human longevity in the **Chinese Han** long-lived individuals and controls. | | | | | | | |
| --- | --- | --- | --- | --- | --- | --- | --- |
| Haplotype | | rs12778366-rs3758391-rs3740051-rs33957861-rs7896005-rs12413112-rs11599176-rs4746720 |  | Frequency | | OR (95% CI) | *P* |
|  | |  |  | Case | Control |  |  |
| 1 | | T-T-A-C-A-G-A |  | 0.3849 | 0.4086 | 1.00 |  |
| 2 | | T-T-G-C-A-G-A |  | 0.2846 | 0.2786 | 1.08 (0.90 - 1.30) | 0.43 |
| 3 | | C-T-A-T-A-A-G |  | 0.1635 | 0.166 | 1.04 (0.83 - 1.30) | 0.73 |
| 4 | | T-C-A-C-G-G-A |  | 0.16 | 0.1416 | 1.19 (0.95 - 1.50) | 0.13 |
| Rare haplotypes | | |  | 0.007 | 0.005 | 1.43 (0.41 - 4.97) | 0.58 |
| Global |  | |  |  |  |  | 0.59 |

| Table S4. Genotype and allele frequencies of *SIRT1* polymorphisms in the long-lived individuals and controls when stratified by gender. | | | | | |
| --- | --- | --- | --- | --- | --- |
| Polymorphisms | Genotype/Allele | Case | Control | OR(95%CI) | *P* |
| Men |  |  |  |  |  |
| rs12778366 | T/T | 73 (71.6%) | 115 (72.3%) | 1.00 | 0.98 |
|  | C/T | 26 (25.5%) | 40 (25.2%) | 1.02 (0.58-1.82) |
|  | C/C | 3 (2.9%) | 4 (2.5%) | 1.18 (0.26-5.43) |
|  | T | 172(84.3%) | 270(84.9%) | 1.00 |  |
|  | C | 32(15.7%) | 48(15.1%) | 1.05 (0.65-1.69) | 0.86 |
| rs3758391 | T/T | 73 (71.6%) | 119 (74.8%) | 1.00 | 0.14 |
|  | C/T | 27 (26.5%) | 40 (25.2%) | 1.10 (0.62-1.94) |
|  | C/C | 2 (2%) | 0 (0%) |  |
|  | T | 173(84.8%) | 278(87.4%) | 1.00 |  |
|  | C | 31(15.2%) | 40(12.6%) | 1.27 (0.75-2.17) | 0.37 |
| rs3740051 | A/A | 43 (42.6%) | 76 (47.8%) | 1.00 | 0.38 |
|  | G/A | 47 (46.5%) | 73 (45.9%) | 1.14 (0.67-1.92) |
|  | G/G | 11 (10.9%) | 10 (6.3%) | 1.94 (0.76-4.95) |
|  | A | 133(65.8%) | 225(70.8%) | 1.00 |  |
|  | G | 69(34.2%) | 93(29.2%) | 1.28 (0.86-1.90) | 0.22 |
| rs33957861 | C/C | 73 (71.6%) | 116 (73%) | 1.00 | 0.96 |
|  | C/T | 26 (25.5%) | 38 (23.9%) | 1.09 (0.61-1.94) |
|  | T/T | 3 (2.9%) | 5 (3.1%) | 0.95 (0.22-4.11) |
|  | C | 172(84.3%) | 270(84.9%) | 1.00 |  |
|  | T | 32(15.7%) | 48(15.1%) | 1.04 (0.65-1.68) | 0.86 |
| rs7896005 | A/A | 73 (71.6%) | 119 (74.8%) | 1.00 | 0.14 |
|  | G/A | 27 (26.5%) | 40 (25.2%) | 1.10 (0.62-1.94) |
|  | G/G | 2 (2%) | 0 (0%) |  |
|  | A | 173(84.8%) | 278(87.4%) | 1.00 |  |
|  | G | 31(15.2%) | 40(12.6%) | 1.27 (0.75-2.17) | 0.37 |
| rs12413112 | G/G | 73 (71.6%) | 115 (72.3%) | 1.00 | 0.98 |
|  | G/A | 26 (25.5%) | 39 (24.5%) | 1.05 (0.59-1.87) |
|  | A/A | 3 (2.9%) | 5 (3.1%) | 0.95 (0.22-4.07) |
|  | G | 172(84.3%) | 269(84.6%) | 1.00 |  |
|  | A | 32(15.7%) | 49(15.4%) | 1.02 (0.64-1.64) | 0.93 |
| rs11599176 | A/A | 73 (71.6%) | 115 (72.3%) | 1.00 | 0.98 |
|  | G/A | 26 (25.5%) | 39 (24.5%) | 1.05 (0.59-1.87) |
|  | G/G | 3 (2.9%) | 5 (3.1%) | 0.95 (0.22-4.07) |
|  | A | 172(84.3%) | 269(84.6%) | 1.00 |  |
|  | G | 32(15.7%) | 49(15.4%) | 1.02 (0.64-1.64) | 0.93 |
| rs4746720 | T/T | 43 (42.2%) | 51 (32.1%) | 1.00 | 0.22 |
|  | C/T | 46 (45.1%) | 80 (50.3%) | 0.68 (0.40-1.18) |
|  | C/C | 13 (12.8%) | 28 (17.6%) | 0.55 (0.25-1.19) |
|  | T | 132(64.7%) | 182(57.2%) | 1.00 |  |
|  | C | 72(35.3%) | 136(42.8%) | 0.73 (0.50-1.05) | 0.086 |
| Women |  |  |  |  |  |
| rs12778366 | T/T | 354 (68.9%) | 463 (67.4%) | 1.00 | 0.59 |
|  | C/T | 143 (27.8%) | 206 (30%) | 0.91 (0.70-1.17) |
|  | C/C | 17 (3.3%) | 18 (2.6%) | 1.24 (0.63-2.43) |
|  | T | 851(82.8%) | 1132(82.4%) | 1.00 |  |
|  | C | 177(17.2%) | 242(17.6%) | 0.97 (0.79-1.21) | 0.8 |
| rs3758391 | T/T | 368 (71.6%) | 501 (73.1%) | 1.00 | 0.8 |
|  | C/T | 134 (26.1%) | 167 (24.4%) | 1.09 (0.84-1.42) |
|  | C/C | 12 (2.3%) | 17 (2.5%) | 0.96 (0.45-2.04) |
|  | T | 870(84.6%) | 1169(85.3%) | 1.00 |  |
|  | C | 158(15.4%) | 201(14.7%) | 1.06 (0.84-1.32) | 0.64 |
| rs3740051 | A/A | 267 (52%) | 360 (52.5%) | 1.00 | 0.74 |
|  | G/A | 211 (41.1%) | 272 (39.6%) | 1.05 (0.82-1.33) |
|  | G/G | 35 (6.8%) | 54 (7.9%) | 0.87 (0.56-1.38) |
|  | A | 745(72.6%) | 992(72.3%) | 1.00 |  |
|  | G | 281(27.4%) | 380(27.7%) | 0.98 (0.82-1.18) | 0.87 |
| rs33957861 | C/C | 352 (68.6%) | 462 (67.2%) | 1.00 | 0.81 |
|  | C/T | 144 (28.1%) | 204 (29.7%) | 0.93 (0.72-1.19) |
|  | T/T | 17 (3.3%) | 21 (3.1%) | 1.06 (0.55-2.04) |
|  | C | 848(82.7%) | 1128(82.1%) | 1.00 |  |
|  | T | 178(17.3%) | 246(17.9%) | 0.96 (0.78-1.19) | 0.72 |
| rs7896005 | A/A | 367 (71.5%) | 504 (73.4%) | 1.00 | 0.77 |
|  | G/A | 134 (26.1%) | 167 (24.3%) | 1.10 (0.85-1.44) |
|  | G/G | 12 (2.3%) | 16 (2.3%) | 1.03 (0.48-2.20) |
|  | A | 868(84.6%) | 1175(85.5%) | 1.00 |  |
|  | G | 158(15.4%) | 199(14.5%) | 1.07 (0.86-1.35) | 0.54 |
| rs12413112 | G/G | 353 (68.7%) | 461 (67.1%) | 1.00 | 0.78 |
|  | G/A | 144 (28%) | 205 (29.8%) | 0.92 (0.71-1.18) |
|  | A/A | 17 (3.3%) | 21 (3.1%) | 1.06 (0.55-2.03) |
|  | G | 850(82.7%) | 1127(82.0%) | 1.00 |  |
|  | A | 178(0.173) | 247(0.18) | 0.96 (0.77-1.18) | 0.67 |
| rs11599176 | A/A | 350 (68.2%) | 461 (67.1%) | 1.00 | 0.86 |
|  | G/A | 146 (28.5%) | 205 (29.8%) | 0.94 (0.73-1.21) |
|  | G/G | 17 (3.3%) | 21 (3.1%) | 1.07 (0.55-2.05) |
|  | A | 846(82.5%) | 1127(82.0%) | 1.00 |  |
|  | G | 180(17.5%) | 247(18.0%) | 0.97 (0.79-1.20) | 0.78 |
| rs4746720 | T/T | 198 (38.5%) | 263 (38.3%) | 1.00 | 1 |
|  | C/T | 225 (43.8%) | 302 (44%) | 0.99 (0.77-1.27) |
|  | C/C | 91 (17.7%) | 122 (17.8%) | 0.99 (0.71-1.38) |
|  | T | 621(60.4%) | 828(60.3%) | 1.00 |  |
|  | C | 407(39.6%) | 546(39.7%) | 0.99 (0.85-1.17) | 0.94 |

| Table S5. Association of *SIRT1* haplotypes with human longevity when stratified by gender. | | | | | | |
| --- | --- | --- | --- | --- | --- | --- |
| Haplotype | rs12778366-rs3758391-rs3740051-rs33957861-rs7896005-rs12413112-rs11599176-rs4746720 |  | Frequency | | OR (95% CI) | *P* |
|  |  |  | Case | Control |  |  |
| Men |  |  |  |  |  |  |
| 1 | T-T-A-C-A-G-A-C |  | 0.3529 | 0.4277 | 1.00 |  |
| 2 | T-T-G-C-A-G-A-T |  | 0.3382 | 0.2893 | 1.49 (0.95 - 2.33) | 0.084 |
| 3 | C-T-A-T-A-A-G-T |  | 0.1569 | 0.1478 | 1.31 (0.77 - 2.22) | 0.32 |
| 4 | T-C-A-C-G-G-A-T |  | 0.152 | 0.1258 | 1.47 (0.83 - 2.58) | 0.19 |
| Rare haplotypes | |  | 0 | 0.0093 |  | 1 |
| Global |  |  |  |  |  | 0.14 |
| Women |  |  |  |  |  |  |
| 1 | T-T-A-C-A-G-A-C |  | 0.393 | 0.3967 | 1.00 |  |
| 2 | T-T-G-C-A-G-A-T |  | 0.2733 | 0.2758 | 1.00 (0.82 - 1.22) | 0.99 |
| 3 | C-T-A-T-A-A-G-T |  | 0.1722 | 0.1761 | 0.99 (0.78 - 1.24) | 0.91 |
| 4 | T-C-A-C-G-G-A-T |  | 0.1537 | 0.1448 | 1.07 (0.84 - 1.36) | 0.59 |
| Rare haplotypes | |  | 0.0079 | 0.0066 | 1.20 (0.36 - 3.95) | 0.76 |
| Global |  |  |  |  |  | 0.97 |

| Table S6. Association studies of *SIRT1* with human longevity | | | | | | | |
| --- | --- | --- | --- | --- | --- | --- | --- |
|  | SNPs | Major>Minor allele | This study | Study by Huang *et al*. | Study by Willcox *et al*. | Study by Flachsbart *et al*. | Study by Kim *et al*. |
| Sample |  |  | 616 cases (≥98 yrs old) and 846 controls (Chinese) | 223 cases (≥90 yrs old) and 227 controls (Chinese) | 213 cases (≥95 yrs old) and 402 controls (Japanese) | 1026 cases (≥95 yrs old) and 547 controls (Caucasians) | 224 cases (≥90 yrs old) and 293 controls replicate:170 cases (≥98 yrs old) and 220 controls (Caucasians) |
| 1 | rs12778366 | T>C | ns |  |  |  |  |
| 2 | rs3758391 | T>C | ns |  |  |  |  |
| 3 | rs3740051 | A>G | ns | ns(rs3740051 and rs2273773) | ns(rs10823112) | ns(rs2273773) |  |
| 4 | rs33957861 | C>T | ns |  |  |  |  |
| 5 | rs7896005 | A>G | ns | ns(rs3758391 and rs10997870) | ns(rs7069102 and rs1885472) | ns(rs3758391, rs1885472 and rs10997870) | **positive allele: A (*P*=5.6×10^-3^)** |
| 6 | rs12413112 | G>A | ns |  |  |  |  |
| 7 | rs11599176 | A>G | ns |  |  |  |  |
| 8 | rs4746720 | T>C | ns | **positive genotype: C/T (*P*<0.001)** |  |  |  |
|  |  |  |  |  |  | ns(rs2234975) |  |
| ns = non-significant association; significant associations (*P*≤0.05) are in bold. | | | | | | | |
